# An immunohistochemical study on the expression of sex steroid receptors, Ki-67 and cytokeratins 7 and 20 in feline endometrial adenocarcinomas

**DOI:** 10.1186/s12917-015-0530-6

**Published:** 2015-08-14

**Authors:** Ana Laura Saraiva, Rita Payan-Carreira, Fátima Gärtner, Marta R. Fortuna da Cunha, Alexandra Rêma, Fátima Faria, Lígia M. Lourenço, Maria dos Anjos Pires

**Affiliations:** CECAV, Centro de Ciência Animal e Veterinária, Universidade de Trás-os-Montes e Alto Douro, Quinta de Prados, 5000-801 Vila Real, Portugal; Escola Universitária Vasco da Gama (EUVG), Avenida José R. Sousa Fernandes, Campus Universitário, Bloco B, Lordemão, 3020-210 Coimbra, Portugal; Abel Salazar Institute of Biomedical Sciences (ICBAS), University of Porto, Rua de Jorge Viterbo Ferreira n.° 228, 4050-313 Porto, Portugal; Institute of Molecular Pathology and Immunology of the University of Porto (IPATIMUP), Rua Júlio Amaral de Carvalho, 45, 4200-135 Porto, Portugal

**Keywords:** Cat diseases, Endometrium, Adenocarcinoma, Immunohistochemistry

## Abstract

**Background:**

Endometrial adenocarcinomas are a rare type of tumour in cats. Though different morphologies have been reported, the most frequent histological type of feline endometrial adenocarcinoma (FEA) is the papillary serous. Characterization of molecular markers expression in FEA may contribute to clarify the pathogenesis of these tumours and to assess the differences between normal endometrium and FEA regarding the expression pattern of several proteins. Therefore, this study aimed to evaluate the immunohistochemical profile of a wide panel of antibodies (specific for ER-α, PR, Ki-67, CK7 and CK20) in twenty-four cases of FEA. Comparisons were made between FEA and feline normal cyclic endometrium in follicular (*n* = 13) and luteal (*n* = 10) stages. Except for Ki-67, all other molecular markers were assessed independently for the intensity of immunolabeling and for the percentage of cells expressing the protein.

**Results:**

This study showed that in FEA a loss of expression occurs for ER-α (*P* ≤ 0.0001) and less markedly also for PR. The lost in sex steroid receptors concerns a decrease in both the proportion of labelled cells and the intensity of immunolabelling (*P* = 0.002 and *P* = 0.024, respectively). Proliferative activity, estimated via Ki-67 immunoreaction, significantly increased in FEA as compared to normal endometrium (*P* ≤ 0.0001). Feline endometrial adenocarcinomas maintained the CK7+/CK20+ status of normal endometrium. However, FEA showed decreased CK7 intensity of labelling compared to normal endometria (*P* ≤ 0.0001) and loss of CK20 expression, both in intensity (*P* ≤ 0.0001) and in percentage of positive cells (*P* = 0.01), compared to normal tissues.

**Conclusions:**

Data gathered in this study suggest that proliferation in FEA accompanies ER-α down-regulation, possibly following activation of pathways mediated by local growth factors. Moreover, FEA retains combined expression of CK7 and CK20, as evidenced in normal endometrial epithelia, although a decrease in CK7 expression was observed.

## Background

Endometrial adenocarcinomas are a rare type of tumour in cats [1–3]. Uterine neoplasms account to 1 to 2 % of the tumours affecting the queen’s reproductive organs, representing 0.2 to 0.4 % of all feline tumours [4]. Nevertheless, in recent years an increasing number of reports on feline endometrial adenocarcinomas (FEA) have been published [5–9], suggesting that FEA may be more common than once believed.

Clinically, FEA are not distinguishable from other non-neoplastic diseases of the cat uterus, like pyometra, though they may have a completely different outcome, particularly in older females [7, 10].

Knowledge on FEA is very restricted and mostly originated from case descriptions, complemented with a few studies developed in a limited case series, supporting the need for additional studies in larger case series [7, 11]. Immunohistochemistry is an acknowledged well-established routine technique in anatomical pathology, very useful on account of its easiness, safety and inexpensiveness compared to other molecular techniques [12]. Moreover, locating a protein in tissue sections may be helpful to study morphological characterization and potential behaviour of tumours.

The cyclic interchange of estrogens and progesterone secreted by the ovaries determines cyclic patterned changes in the mammalian endometrium – the endometrial cycle - with the ultimate goal of achieving a pregnancy. In the endometrium, major functions of circulating sex steroids are dependent on the estrogen and progesterone receptors (ER and PR). Particularly, these receptors mediate the continuous synchronized epithelial-stromal crosstalk that ultimately regulates the endometrial proliferation, differentiation and secretion and thereby promote embryo receptivity [13, 14]. In general terms, estrogen stimulates the proliferation of glandular and stromal cells, whereas progesterone inhibits the growth of glandular cells and stimulates the secretory activity in the endometrial glands. A disruption in the equilibrium of ER/PR [15], or mutations in the genes coding these molecules [16], may interfere with the normal proliferative or secretory patterns, and predispose to endometrial disease. Estrogen and progesterone receptors have been described in human endometrial carcinomas as independent prognostic factors [17]. Information on sex steroid receptors in feline endometrium is scarce. Furthermore, in FEA, available data concerning sex steroid receptors expression is limited and frequently opposed. In general, loss of ER-α has been reported, ranging from 50 % (4/8 cases) [18] to 83.3 % (5/6 cases) [11]; one study also refers that PR are generally expressed in FEA [11]. This may raise an important concern when sub-clinical FEA females are under progestagen contraceptive treatment, which could interfere with FEA progression and outcome. Moreover, the hormone receptors status of FEA may adjoin important information for medical management after ovariohysterectomy (OVH) [11], deserving additional studies.

In general, cancer development and progression is associated with deregulation of cell proliferation and of programmed cell death. The increased proliferative activity in a tumour is related to its growth rate, and may account for its malignancy and the clinical course of the disease. Thus, its assessment yields useful prognostic information related to survival of patients in various types of tumours [19]. Evaluation of the tumour proliferative activity is frequently assessed by immunohistochemistry, using the expression of Ki-67 nuclear antigen [20], a nuclear non-histone protein present exclusively in proliferating cells, whether they are normal or neoplastic [21]. Assessment of Ki-67 index has been applied to the normal endometrium, to characterize the cyclic changes in cell proliferation in mares [22], cows [23] and bitches [24]. Furthermore, the immunohistochemical profile of Ki-67 has also been sporadically determined in feline endometrial lesions, including in two FEA case reports [8] - in which Ki-67 varied from moderate to high - and in the description of multiple uterine lesions, which included an area of endometrial adenocarcinoma, that exhibited 40 % of positive cells for Ki-67 [25].

Cytokeratins (CK) are the largest group of intermediary filaments proteins; they are essential in the development and differentiation of epithelial cells. They are also crucial for the normal structure and function of the epithelium, as CK are involved in signal transduction, cell polarity and gene regulation [26]; in addition, particular CK may also contribute to the epithelial innate defence mechanisms, through their antimicrobial properties [27]. Cytokeratins are divided into two groups. Cytokeratin 20 is included in the type I CK, which are acidic, low molecular weight (40–56.5 kDa) proteins; whilst CK7 belongs to type II CK, that consist of basic, high molecular weight (52–67 kDa) proteins [28]. Different types of epithelia show specific patterns of CK expression. In the uterus, CK are commonly found in the luminal and glandular epithelia [26] and in the invading trophoblast [29, 30]. Antibodies raised against CK are used as specific markers for epithelial cell differentiation and are largely used for tumour identification and classification [31]. The human uterine carcinoma presents a CK7+/CK20- phenotype [26]. Espinosa de los Monteros et al. (1999) strengthen the usefulness of the coordinate expression of CK7 and CK20 to distinguish different primary feline carcinomas and to ascertain its origin, in case of metastatic disease; they also described the normal pattern of these CK in feline normal endometrium [32]. Cytokeratin 7+/cytokeratin 20+ profiles were described in 2/3 (66.7 %) FEA [32]. In another study, 3/6 (50.0 %) FEA expressed CK7, whereas 4/6 (66.7 %) FEA showed positive reaction for CK20 [18].

Most published case series studies on FEA used a small number of cases, ranging from three [32] to six [11] or eight [18], which might have contributed to the reported contrasting results.

Therefore, the objectives of this study were: 1) to evaluate the immunohistochemical expression of ER-α, PR, Ki-67, CK7 and CK20 in the papillary serous form of FEA using the largest case series reported so far; 2) to monitor the changes in the immunoexpression of these molecules as compared to the immunohistochemical profile of feline normal endometrium in two different stages of the estrous cycle; 3) to estimate putative associations between the molecular markers and the histopathological predictors of dedifferentiation; 4) to study the relationship between these markers.

## Methods

### Samples and animals

The study was conducted in twenty-four samples of FEA retrieved from the archives of four different laboratories, during a period of 8 years. As controls, twenty-three archived samples of histologically normal feline uteri were selected (13 samples for the follicular stage – FS – and 10 samples for the luteal stage – LS). All samples were previously fixed in 4 % neutral-buffered formalin and routinely processed for paraffin embedding.

Control uterine samples were obtained after elective ovariohysterectomy (OVH), from post-pubertal queens aged seven months to eight years of age (mean 1.5 years). Breed was unavailable in 65.3 % of the records; on the other 34.7 % records, represented breeds included Domestic Shorthaired cats (*n* = 7; 30.4 %) and Persian (*n* = 1; 4.3 %). For controls (normal endometria), only queens not submitted to contraceptive treatment were selected.

Feline endometrial adenocarcinomas were diagnosed in queens aged one year to 15 years of age (mean 7.9 years); breeds included Domestic Shorthaired cats (*n* = 17; 70.8 %), Siamese (*n* = 2; 8.3 %) and Persian (*n* = 1; 4.2 %). Contraception was given in five (20.8 %) animals and was denied in three (12.5 %) FEA cases, though the length of treatment was not mentioned in the form; no information existed in the request form for the remainder 16 cases (66.7 %).

Regarding the clinical history of the animals diagnosed for FEA, data was collected from the histopathological request forms. The existence of clinical signs of uterine disease was mentioned in 11 (45.8 %) cases, whilst in six (25.0 %) other cases, the coexistence of pyometra and a concurrent mammary tumour were referred. FEA was diagnosed in two (8.3 %) animals without acknowledge clinical symptoms, the lesions in the uterus being detected only during elective OVH as an enlarged organ with increased consistency. For all the other cases (*n* = 5; 20.8 %), the reasons for OVH were not declared.

### Ethics statement

The study was approved by the Ethics Committee of University of Trás-os-Montes and Alto Douro (Vila Real, Portugal), permission number DOC22/CE/2014. None of the animals was subjected to OVH purposely for the present study.

### Morphological evaluation

Feline endometrial adenocarcinomas diagnosis and the staging of the cycle stage in normal samples of healthy endometria (controls) were performed by light microscopy, on three-micrometres sections routinely stained with haematoxylin and eosin. The tumours were evaluated according to several criteria of malignancy described in the literature [18, 33, 34], enabling the diagnosis of FEA of the papillary serous type [35]. The histopathological features included: nuclear atypia, classified as low to moderate or high; mean number of mitoses per high power field (HPF), scored as lower than one, one to five and more than five; and the existence of myometrium, serosa or vascular/lymphatic invasion, evaluated as present or absent.

Normal uterine samples were staged as FS or LS based on the summative information gathered by the ovarian morphology (presence of follicles *vs.* corpora lutea), and the histological endometrial features (the epithelial cell height and the degree of development and coiling of endometrial glands). For patients diagnosed with FEA, determination of the stage of the estrous cycle was evaluated according to the presence of follicles in different stages of development – FS – or the presence of corpora lutea – LS – in the ovary.

For FEA cases, 11 cats (45.8 %) were in FS and seven animals (25.0 %) were in the LS of the estrous cycle; on the remaining cases (*n* = 7; 29.2 %), the surgical specimen did not include the ovaries thus impairing the staging of the estrous cycle.

### Immunohistochemistry

Immunohistochemistry was performed in three-micrometre sections by the indirect avidin-biotin peroxidase complex technique. Table 1 summarizes the references of the antibodies used in this study, their dilution and incubating time. Antigen retrieval was performed in a steamer with slides immersed in boiling citrate buffer (pH 6.0; about 94 °C) for 3 min. After cooling in phosphate buffered saline (PBS), the sections were immersed in 3 % hydrogen peroxide during 20 min to block endogenous peroxidase activity. After the incubation with the normal serum for 5 min, the slides were incubated with the primary antibodies (Table 1) for an overnight period, in a humid chamber. Immunohistochemical labelling was achieved by using the products specified in Table 1, following the manufacturer’s instructions. Colour was developed with 3, 3-diaminobenzidine tetrahydrochloride and sections were counterstained with Gill’s haematoxylin, dehydrated and mounted for evaluation on light microscopy.

**Table 1:** Primary antibodies used for immunohistochemistry

Antibody	Clone	Source	Dilution	Staining pattern	Labelling
Monoclonal mouse anti-human estrogen receptor (ER)	ER-12	Cell Marque®, USA	1:40	Nuclear	Novolink® Polymer Detection System, (RE7280-K) Leica Biosystems®, UK
Monoclonal mouse anti-human progesterone receptor (PR)	1A6	Novocastra®, UK	1:30		
Monoclonal Mouse anti-human Ki-67 Antigen	MIB-1	Dako®, Denmark	1:50		
Clonal rabbit anti-human Cytokeratin 7 (CK7)	R17-S	DB Biotech®, Slovak Republic	1:100	Cytoplasm	Lab Vision® UltraVision® Large Volume Detection System, Thermo Fisher Scientific®, USA
Monoclonal mouse anti-human Keratin 20 (CK20)	Ks 20.8	Thermo Fisher Scientific®, USA	1:100		

### Quantification of immunolabelling

In normal endometrium, the immunolabelling for ER-α, PR, Ki-67, CK7 and CK20 was evaluated independently in the surface epithelium (SE), superficial and deep glandular epithelium (SGE and DGE, respectively). In FEA the immunostaining was assessed in epithelial tumour cells. Stromal and myometrial labelling were evaluated independently for ER-α and PR in both normal and neoplastic epithelium.

The intensity of ER-α and PR immunolabelling was graded as 0 = no staining, 1 = weak, 2 = moderate and 3 = strong. Regarding the percentage of cells expressing ER-α and PR, the negative cut-off was established at 5 % [12, 36–38]. Since the majority of the controls (*n* = 20/23) had more than 80 % of positive cells, this was settled as the maximum cut-off. Therefore, the samples were further classified semiquantitatively according to the marks: 0 = negative (≤5 % positive nuclei); 1 = loss of expression (5 to 80 % positive nuclei) and 2 = positive (≥80 % positive nuclei).

The evaluation of Ki-67 immunostaining was performed in 1000 cells in 10 HPF (x 400) and expressed as a percentage – proliferative index [20].

The immunoexpression for CK7 and 20 was semiquantitatively scored for both the percentage of labelled cells (1 to 33 % = low; 34 to 66 % = moderate; 67 to 100 % = high) and the labelling intensity (1 = weak; 2 = moderate; 3 = strong) [32]. This evaluation was performed for the entire endometrium section in controls and in representative microscopic fields for FEA. The labelling intensity was evaluated on the basis of the most frequently observed.

### Statistical analysis

For data concerning the sex steroids and CK immunolabeling (categorical variables) the statistical comparisons were performed by using chi-square and Fisher exact tests in the IBM SPSS Statistics Base 20.0 software®. Ki-67 data were analysed using the ANOVA test, the post hoc paired comparisons were carried out using the Bonferroni correction. *P* values < 0.05 were regarded as statistically significant.

## Results

### Histopathological evaluation

**Fig. 1:**
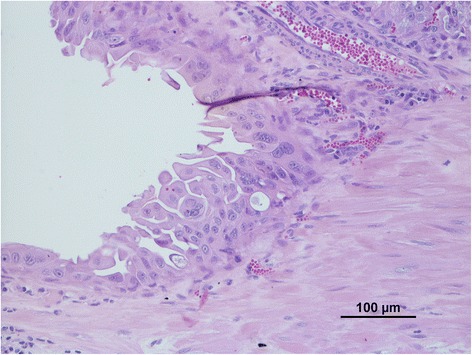
FEA. Papillary growth of high atypical epithelial tumour cells, invading uterine myometrium. Haematoxylin and eosin. BAR = 100 μm

In the present study, FEA were primarily characterized by the multi-layered proliferation of neoplastic endometrial epithelial cells on papillae into the lumen supported by a thin fibrovascular stroma. Tubular and solid proliferation was scantly present. Therefore, tumours were histologically classified as FEA of the papillary serous type (Fig. 1). Neoplastic cells were pleomorphic columnar shaped, with a moderate amount of eosinophilic cytoplasm and round-to-oval, vesicular or hyperchromatic nuclei that lost the normal polarity. Numerous multinucleated cells with darkened nuclei were present within and at the surface of the lesions. A variable number of clear cells - large, round to polygonal cells, with foamy cytoplasm and eccentric, crenated or ovoid nucleus – comprised less than 50 % of the tumours’ area. Nucleoli were evident; occasional intranuclear clear inclusions were also found. Randomly distributed areas of necrosis within the tumours were frequently present. A variable degree of atypia was found in FEA lesions (Table 2), with 54.2 % (13/24) of the cases evidencing high atypia.

**Table 2:** Main histological features of feline endometrial adenocarcinomas in comparison to the normal endometrium

		Atypia			Number of mitoses HPF			Myometrial invasion	Serosa invasion	Vascular/lymphatic invasion
		n (%)			n (%)					
		Absent	Low to moderate	High	≤1	1-5	≥5			
								n (%)	n (%)	n (%)
FS	SE	13 (100.0)	0	0	13 (100.0)	0	0	0	0	0
	SGE	13 (100.0)	0	0	12 (92.3)	1 (7.7)	0	0	0	0
	DGE	13 (100.0)	0	0	12 (92.3)	1 (7.7)	0	0	0	0
LS	SE	10 (100.0)	0	0	10 (100.0)	0	0	0	0	0
	SGE	10 (100.0)	0	0	10 (100.0)	0	0	0	0	0
	DGE	10 (100.0)	0	0	7 (70.0)	3 (30.0)	0	0	0	0
FEA		0	11 (45.8)	13 (54.2)	3 (12.5)	19 (79.2)	2 (8.3)	16 (66.7)	1 (4.2)	3 (12.5)

*FS* = follicular stage; *LS* = luteal stage; *FEA* = feline endometrial adenocarcinomas; *SE* = surface endometrium; *SGE* = superficial glandular epithelium; *DGE* = deep glandular epithelium; *HPF* = high power field

The mean number of mitoses per HPF in FEA was higher, compared to normal epithelia (*P* ≤ 0.0001). In the majority of FEA (*n* = 19; 79.2 %), the mean number of mitoses per HPF was established between one and five, with very few cases (*n* = 2; 8.3 %) presenting more than five mitoses per HPF (Table 2), in contrast with the observed in the normal endometrium. The SE always presented less than one mitosis per HPF, both whether the FS and LS was considered (Table 2). The mean number of mitoses per HPF in the glandular epithelia was more variable, but the prevailing value was less than one for the SGE in 92.3 % (12/13) to 100 % (10/10) of the samples in FS and LS, respectively. The mean number of mitoses was similar in the DGE in the FS (92.3 %; 12/13), but slightly lower in the LS (70.0 %; 7/10) (Table 2).

Myometrial invasion was observed in a large proportion of cases (66.7 %; 16/24), while vascular invasion was observed in only 12.5 % (3/24) of the cases; serosa impairment was only detected in 4.2 % (1/24) FEA; though vascular and serosa invasion occurred independently in either situation myometrial invasion. In normal uterine samples, as expected, the anatomical integrity of myometrium and serosa layer was maintained.

### Immunohistochemistry

**Fig. 2:**
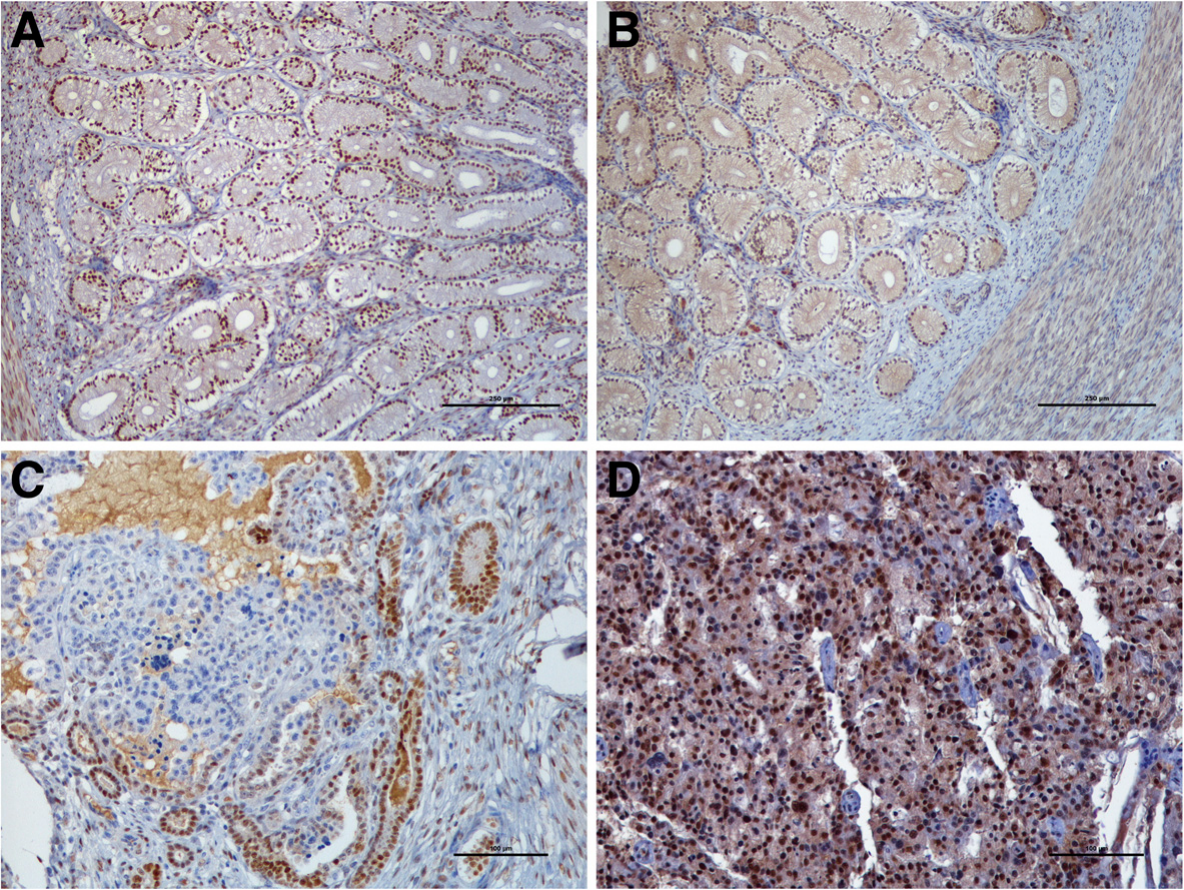
ER-α and PR immunohistochemical expression in feline normal and neoplastic endometrium. **a**. ER-α and **b**. PR in the LS of cyclic endometrium are expressed in all uterine layers. BAR = 250 μm **c**. ER-α expression is decreased in FEA. Transition between positive normal glands and negative neoplastic cells are notorious. BAR =100 μm. **d**. PR expression is maintained in FEA. BAR =100 μm. Counterstained with Gill’s haematoxylin

In the samples of normal endometrium, immunoreaction against ER-α and PR was consistently detected for all epithelial types, as well as for the stroma and myometrium (Table 3; Fig. 2 (a-b)).

**Table 3:** Results for the immunoexpression of the ER-α and PR in the normal feline uterus (at the FS and LS) and in the neoplastic epithelium in FEA

		Intensity				Percentage of positive cells		
		n (%)				n (%)		
		0	1	2	3	0	1	2
		ER-α						
FS	SE	0	0	7 (53.8)	6 (46.2)	0	0	13 (100.0)
	SGE	0	0	3 (23.1)	10 (76.9)	0	0	13 (100.0)
	DGE	0	0	1 (7.7)	12 (92.3)	0	0	13 (100.0)
	Stroma	0	0	4 (30.8)	9 (69.2)	0	7 (53.8)	6 (46.2)
	Myometrium	0	0	12 (92.3)	1 (7.7)	0	0	13 (100.0)
LS	SE	0	3 (30.0)	5 (50.0)	2 (20.0)	0	2 (20.0)	8 (80.0)
	SGE	0	0	6 (60.0)	4 (40.0)	0	0	10 (100.0)
	DGE	0	0	0	10 (100.0)	0	0	10 (100.0)
	Stroma	0	1 (10.0)	4 (40.0)	5 (50.0)	0	7 (70.0)	3 (30.0)
	Myometrium	0	1 (10.0)	7 (70.0)	2 (20.0)	0	0	10 (100.0)
FEA	Epithelium	12 (50.0)	5 (20.8)	6 (25.0)	1 (4.2)	12 (50.0)	11 (45.8)	1 (4.2)
	Stroma	11 (45.8)	2 (8.3)	8 (33.3)	3 (12.5)	11 (45.8)	13 (54.2)	0
	Myometrium	4 (16.7)	0	18 (75.0)	2 (8.3)	4 (16.7)	9 (37.5)	11 (45.8)
		PR						
FS	SE	0	1 (7.7)	7 (53.8)	5 (38.5)	0	0	13 (100.0)
	SGE	0	1 (7.7)	7 (61.5)	5 (30.8)	0	0	13 (100.0)
	DGE	0	2 (15.4)	5 (38.5)	6 (46.2)	0	0	13 (100.0)
	Stroma	0	1 (7.7)	10 (76.9)	2 (15.4)	0	10 (76.9)	3 (23.1)
	Myometrium	0	5 (38.5)	8 (61.5)	0	0	0	13 (100.0)
LS	SE	0	0	5 (50.0)	5 (50.0)	0	1 (10.0)	9 (90.0)
	SGE	0	0	5 (50.0)	5 (50.0)	0	1 (10.0)	9 (90.0)
	DGE	0	1 (10.0)	3 (30.0)	6 (60.0)	0	1 (10.0)	9 (90.0)
	Stroma	4 (40.0)	1 (10.0)	2 (20.0)	3 (30.0)	4 (40.0)	6 (60.0)	0
	Myometrium	1 (10.0)	4 (40.0)	3 (30.0)	2 (20.0)	1 (10.0)	2 (20.0)	7 (70.0)
FEA	Epithelium	0	1 (4.2)	19 (79.2)	4 (16.7)	0	7 (29.2)	17 (70.8)
	Stroma	2 (8.3)	1 (4.2)	19 (79.2)	2 (8.3)	2 (8.3)	21 (87.5)	1 (4.2)
	Myometrium	0	4 (16.7)	18 (75.0)	2 (8.3)	0	6 (25.0)	18 (75.0)

*SE* = surface endometrium; *SGE* = superficial glandular epithelium; *DGE* = deep glandular epithelium; *FS* = follicular stage; *LS* = luteal stage.). Intensity of immunolabelling: 1 = weak, 2 = moderate and 3 = strong. Percentage of positive nuclei: 0 = negative (≤5 % positive nuclei); 1 = loss of expression (5-80 % positive nuclei) and 2 = positive (≥80 % positive nuclei)

The intensity of labelling against ER-α was, in general, weaker in the SE than in the GE and the intensity scores were higher in the DGE than in SGE. However, the differences in the represented epithelia were significant for LS, but not for FS (*P* = 0.011 and *P* = 0.593, respectively; Table 3). The intensity of immunostaining for ER-α was slightly lower in the SE and SGE during LS in comparison to FS, but changes were more discrete regarding the DGE intensity of immunostaining. A moderate to strong intensity (scores 2 or 3) was evidenced in both the stroma and the epithelial elements in the normal endometrium for most samples (Table 3). A slight reduction was observed in the ER-α intensity for the endometrial stroma (Table 3). However, the stage of the cycle did not significantly affect the intensity of ER-α expression in stroma (*P* = 0.507). In the myometrium, the moderate intensity of ER-α staining prevailed in both stages, with no differences detected between the FS and LS (*P* = 0.363).

Regarding the percentage of positive cells for ER-α, all the represented endometrial epithelia expressed this marker during FS (score 2 for all samples; Table 3). A slight reduction was observed in the percentage of labelled cells in the SE of LS, but not in the other endometrial epithelia (Table 3); still, the majority of samples retained a score 2 in the SE of LS, and the differences were non-significant. In FS, the number of cells ER-α positive in the endometrial stroma was more heterogeneous than in the endometrial epithelia, the scores ranging between 1 (5 to 80 % of positive nuclei) and 2 (score 2 ≥ 80 % of positive nuclei). The stromal compartment in LS showed a reduction in the number of ER-α positive cells as compared to the FS, but these changes were not significant (*P* = 0.669). The myometrium was consistently positive to ER-α expression, independently of the stage of the cycle (Table 3).

In what regards the ER-α immunoreaction in FEA, a marked decrease in the intensity and the percentage of labelled cells was recorded for both the epithelium and the stromal compartments (Fig. 2c). In FEA, around 70 % of the samples presented a score ≤ 1 for epithelial ER-α intensity, considerably lower than any epithelia in either the FS or LS normal endometrium (*P* ≤ 0.0001; Table 3). Simultaneously, a marked decrease in the percentage of positive cells to ER-α in the neoplastic epithelium was observed as compared to normal endometrium epithelia (*P* ≤ 0.0001): in FEA, half the samples showed positive nuclei in less than 5 % of the cells, with 45.8 % of the cases displaying ER-α positive nuclei in less than 80 % of the cells (Table 3). Similarly, a loss in the overall expression of ER-α, both in intensity of the immunoreaction and the number of cells with positive nuclei, was observed in FEA stromal compartment compared to the normal endometrial stroma (*P* ≤ 0.0001; Table 3). Tumours characterized by myometrial invasion were more likely to be negative for ER-α in the stromal compartment (*P* = 0.033). The percentage of ER-α positive smooth muscle cells in the FEA myometrium was considerably lower than in the normal endometrium (*P* ≤ 0.0001); notwithstanding, the intensity of the ER-α immunoreaction did not change between normal endometrium and FEA (*P* = 0.153).

In general, the intensity of PR immunoreaction was similar between all the represented endometrial epithelia (Table 3). In the FS a moderate intensity prevailed over the strong intensity of immunolabeling, particularly in the SE and SGE (Table 3). The intensity of immunostaining showed a slight increase during the LS in all the represented epithelia, in particular in the DGE, but these changes were devoid of significance. The immunoreaction in the stromal compartment was more heterogeneous than the observed for ER-α. Higher scores were recorded in the FS; in LS it was observed a marked loss in the intensity of immunostaining (*P* = 0.010; Table 3). Contrasting, lower scores for PR intensity of immunostaining were observed in myometrium in FS compared to LS, but in the latter a wider variation of intensity scores was obtained. However, the differences among FS and LS were non-significant for this layer (*P* = 0.141).

Progesterone receptors were consistently positive in all evaluated epithelia during the FS; in comparison, a slight non-significant decrease in the percentage of PR positive cells was observed in LS (*P* = 0.435; Table 3). The percentage of PR positive cells in the stromal compartment was higher in the FS than in the LS (*P* = 0.027; Table 3). However, score 1 (5 to 80 % of positive nuclei) was the most prevalent in both stages. A small non-significant reduction in the percentage of PR positive cells was observed also in myometrium during the LS, compared to FS (*P* = 0.070; Table 3).

Compared to the normal endometrium, FEA displayed more discrete differences in what concerns PR expression than those presented for ER-α, both at the epithelial and the stroma compartment (Table 3; Fig. 2d). Feline endometrial adenocarcinomas epithelium showed a significant reduction in either the percentage of PR positive of cells (*P* = 0.002) and the intensity of immunolabeling (*P* = 0.024). Albeit a small decrease in the intensity and the percentage of cells with positive nuclei in both FEA stroma and myometrium compared to normal endometrium, the differences in PR expression in these compartments were devoid of significance (Table 3).

No association between ER-α and PR expression was found in the epithelial or stromal compartments of normal or neoplastic endometria. Also, we did not find a significant association between hormone receptor status and the stage of the estrous cycle in FEA.

**Fig. 3:**
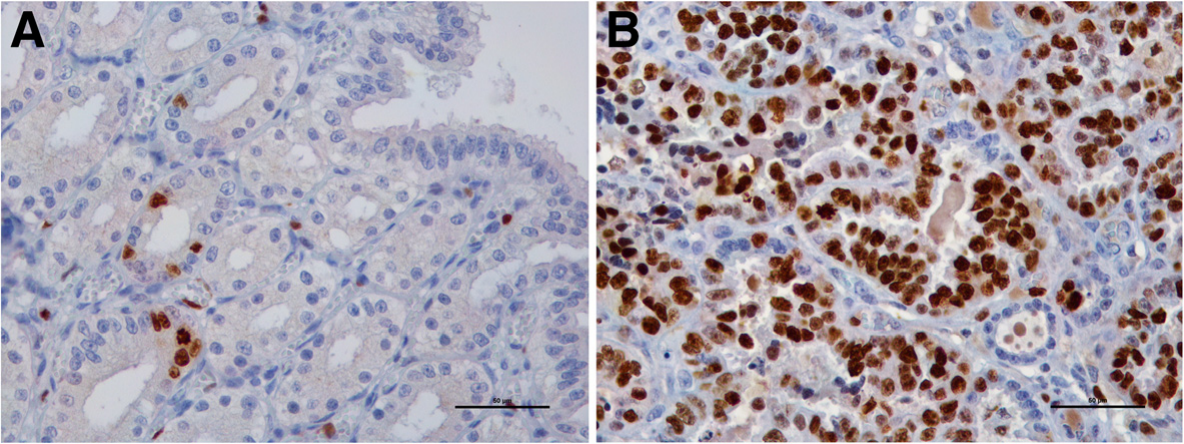
Ki-67 immunohistochemical expression in feline endometrium. **a**. Normal endometrium at the LS shows scarcely positive c ells. A positive cell in metaphase is positive to Ki-67 antigen at the lower bottom. **b**. FEA are largely positive to Ki-67 antigen. BAR = 50 μm. Counterstained with Gill’s haematoxylin

The proliferative indexes, as estimated by Ki-67 counting, were similar between FS and LS (*P* > 0.05). In FS, the proliferative indexes were higher for SGE (16.7 ± 5.4) as compared to SE and DGE (9.0 ± 2.6 and 7.3 ± 2.8, respectively). In LS, the proliferative indexes were higher for the glandular epithelia, particularly for the DGE (21.5 ± 10.0 *vs.* 13.0 ± 5.2 in SGE), than for the SE (7.1 ± 2.1). Considering the epithelia as a whole, the mean proliferative indexes were 11.0 ± 2.3 and 13.9 ± 3.8 in FS and LS, respectively. The proliferative index was considerably higher in the neoplastic epithelium (42.9 ± 3.8) than in normal endometrial epithelia in FS (95 % CI = 20.9 – 42.9) or LS (95 % CI = 17.3 – 40.8) (Fig. 3). Ki-67 expression was independent of the tested clinicopathological features analysed as an indication of tumour aggressiveness and of the hormonal receptor status.

**Fig. 4:**
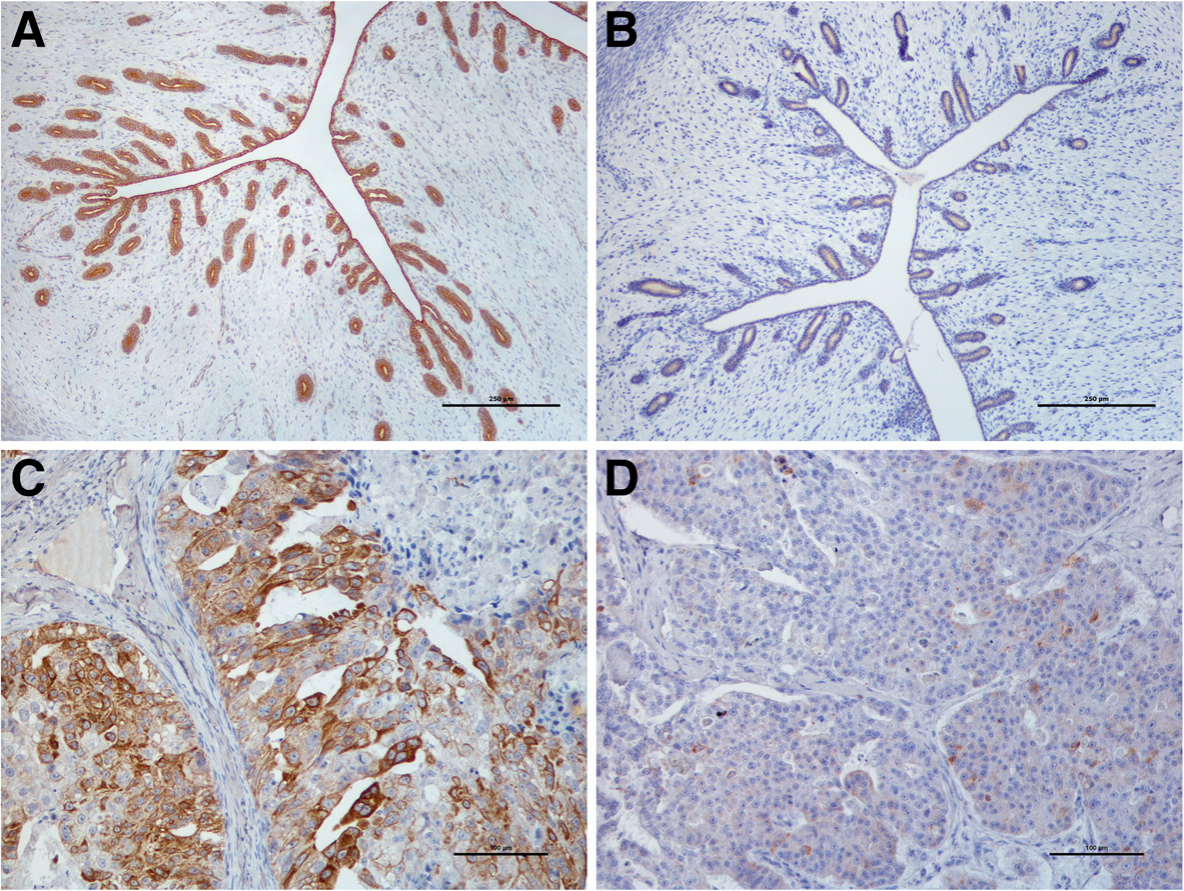
CK7 and CK20 immunohistochemical expression in feline endometrium. **a**. CK7 is strongly expressed in all epithelia of normal endometrium in FS. Contrasting, **b**. CK20 is expressed with a low intensity of labelling. BAR = 250 μm. **c**. FEA displays a maintenance of CK7+ expression although with a heterogeneous positivity **d**. CK20+ immunophenotype in FEA shows a decreased expression and a scarcely, heterogeneous positivity. BAR = 100 μm. Counterstained with Gill’s haematoxylin

Normal feline endometrium presented a CK7+/CK20+ immunoprofile (Fig. 4a-b). The SE presented a strong intensity of immunoreaction against CK7, which did not change with the stage of the estrous cycle (Table 4). The intensity of CK7 immunolabeling differed between the SGE and the DGE, according to the stage of the cycle. A strong intensity of immunolabelling prevailed in the SGE and in the DGE in FS, but a decrease in the labelling intensity for this molecule was observed in both epithelia during the LS (*P* = 0.04 and *P* = 0.039, respectively for SGE and DGE; Table 4), whereby the most prevalent intensity of labelling was the moderate. Cytokeratin 7 was consistently detected by all the epithelia represented in the endometrium, independently of the stage of estrous cycle (Table 4).

**Table 4:** Results for the CK 7 and 20 immunolabelling in the epithelial cells from normal feline endometrium (at the FS and LS) and in FEA

		Intensity			Percentage of positive cells		
		n (%)			n (%)		
		Low	Moderate	High	1	2	3
		CK7					
FS	SE	0	0	13 (100.0)	0	0	13 (100.0)
	SGE	0	0	13 (100.0)	0	0	13 (100.0)
	DGE	0	2 (15.4)	11 (84.6)	0	0	13 (100.0)
LS	SE	0	0	10 (100.0)	0	0	10 (100.0)
	SGE	0	7 (70.0)	3 (30.0)	0	0	10 (100.0)
	DGE	0	6 (60.0)	4 (40.0)	0	0	10 (100.0)
FEA	Epithelium	6 (25.0)	14 (58.3)	4 (16.7)	0	2 (8.3)	22 (91.7)
		CK20					
FS	SE	4 (30.8)	9 (69.2)	0	0	0	13 (100.0)
	SGE	2 (15.4)	11 (84.6)	0	0	0	13 (100.0)
	DGE	1 (7.7)	10 (76.9)	2 (15.4)	0	0	13 (100.0)
LS	SE	0	0	10 (100.0)	0	2 (20.0)	8 (80.0)
	SGE	1 (10.0)	5 (50.0)	4 (40.0)	0	0	10 (100.0)
	DGE	3 (30.0)	7 (70.0)	0	0	0	10 (100.0)
FEA	Epithelium	8 (33.3)	8 (33.3)	8 (33.3)	0	13 (54.2)	11 (45.8)

*SE* = surface endometrium; *SGE* = superficial glandular epithelium; *DGE* = deep glandular epithelium; *FS* = follicular stage; *LS* = luteal stage. Intensity of immunolabelling: 1 = weak, 2 = moderate and 3 = strong

The stage of the cycle affected the intensity of immunoexpression for CK20. The intensity of immunostaining most often recorded in FS was the moderate, the DGE presenting a slightly increased immunostaining as compared with the SE and the SGE (Table 4). In LS, a shift towards stronger intensities was observed in the SE (*P* = 0.002) and in the SGE (*P* = 0.045), but not in the DGE (Table 4). As for the percentage of positive CK20 cells, similar scores were observed in FS and LS, despite the small non-significant decrease observed in the surface epithelium in the LS (*P* = 0.178; Table 4).

In FEA the CK7+/CK20+ epithelial immunoprofile was maintained (Fig. 4c-d). However, a heterogeneous, patchy immunolabelling was observed for both CK in the neoplastic epithelium. A loss in CK7 expression intensity was observed in FEA as compared to the normal endometrial epithelium (*P* ≤ 0.0001), whilst the percentage of CK7 positive cells remained practically unchanged (*P* = 0.065; Table 4). Similarly, CK20 was also lost in FEA as compared to the normal endometrium, both in terms of the percentage of positive cells and the intensity of labelling (*P* ≤ 0.0001 and *P* = 0.01, respectively; Table 4). No relation was established between CK7 and CK20 status in epithelial cells.

The comparison between the immunohistochemical results and the available clinicopathological data suggested that the myometrial invasion observed in FEA was associated with negative stromal ER-α status (*P* = 0.033 and *P* = 0.006, respectively for percentage of positive cells and intensity of immunolabelling) and with a higher percentage of CK20-positive cells (*P* = 0.033). In tumours, nuclear atypia was related to a lower intensity of CK7 labelling (*P* = 0.026). The loss of PR positive cells in the myometrium in FEA was related to a higher nuclear atypia in carcinoma cells (*P* = 0.016).

## Discussion

Despite the rarity of FEA described in the literature [1–3], a recent increasing number of reports suggest that the prevalence of these tumours may be underestimated [5–8]. Moreover, in one study on uterine tumours in domestic cats, FEA was the most commonly diagnosed neoplasm [18]. The selection of cases diagnosed as FEA from the archives of four different laboratories allowed the use of a larger series than usual. Its architecture and the histological features of neoplasic epithelial cells classified the cases under papillary serous type, the most frequent type of FEA [35]. Herein, we describe an immunohistochemical panel performed on FEA to gather helpful information regarding its diagnosis and management, as well as to drive upcoming areas for study on FEA.

Estrogen receptor alpha and PR expression was found in epithelial and stromal endometrial compartments and in myometrium of normal feline uteri. Information on ER-α and PR expression in normal healthy endometrium of this species is very limited, and mostly based on the work of Li et al. (1992) that mimicked the ovarian steroids effects on the feline uterus through the scheduled administration of exogenous estrogens and progesterone [39]. However, changes in the intensity of immunostaining of ER-α from FS to LS followed the expected physiological modifications, evidencing a small decrease in the LS suggestive of the suppressive effect of progesterone receptor activation. Changes were more notorious in the surface epithelium and the superficial glandular epithelia than in deep glandular epithelium; this may be related with to the increased branching of upper endometrial glands in FS and to the persistency of proliferation of basal glands during the LS, that is reflected in the increased coiling reported in this stage [40], which was supported by data gathered by Ki-67 immunolabeling.

Progesterone receptor expression in epithelial cells from normal endometrium showed a small decrease from the FS to LS, following an expectable physiological pattern. The high heterogeneity of the intensity scores presented by the different samples might relate to individual differences in the moment of the LS or the blood levels of progesterone, which were not assessed in the present study. The unavailability of information concerning the normal PR expression in cat endometrium limits the interpretation of the decrease in the PR stromal expression during LS. However, it is possible that, in line with other species, stromal and epithelial compartments of the endometrium may respond differently to steroid hormones [22]. In addition, since endometrial stroma and epithelium influence each other proliferation and differentiation [41], differences in either compartment responses to sex steroids may be necessary to the normal interplay through the uterine cycle.

Recently, a consensus was proposed on the standard guidelines for hormone receptor assessment using immunohistochemistry for canine mammary tumours [42]. However, no guidelines exist for feline mammary or uterine neoplasms. Consequently, the cases used in the present study were evaluated on the basis of the results obtained in controls, using the negative cut-off established before for feline mammary tumours and human endometrial carcinomas [12, 36–38].

All FEA analysed herein lost ER-α immunoexpression in comparison to the normal endometrium. Moreover, the tumours were negative for epithelial and for stromal expression of ER-α (respectively in 50.0 % and 45.8 % of the samples).

The role of estrogen receptors in the regulation of mammalian endometrium, particularly the endometrial proliferation, remains unclear. Uterine proliferation seems to depend on ER mediated transcription, which may result from either the ligand ER-α activation (associated to estrogen stimulation) or a ligand-independent pathway [43]. Activation of the ER-α drives the transactivation of numerous growth factors, which in turn activate their cognate receptors, leading to multiple signalling cascades controlling cellular proliferation [44]. Estrogen receptor alpha may be induced in estrogen-driven tumours, and tumour growth is often limited by progesterone, once ER expression is down-regulated by activated PR [44]. However, proliferation in a tumour may occur driven by the constitutive activation of a parallel growth factor pathway. In that case, proliferation would not depend on the presence of estrogen and progesterone.

Down-regulation of ER-α expression in a variety of tissues has been associated to methylation and to loss of transcriptional activators [45, 46] or to transdominance by ER-β which has an anti-proliferative role [13, 47]. These would explain the loss of ER expression and the acquisition of a hormone resistance status [46], often associated with high-grade adenocarcinoma developing in the uterus. Such independence from the sex steroid control is considered a negative indicator for the clinical outcome [44].

It was shown that in the endometrium, the loss of ER-α compromises E2-induced VEGF expression in epithelial cells, shifting VEGF production to stromal cells thereby inducing stroma-mediated epithelial cell proliferation [48]. Data from the present study suggest that FEA may enter the category of tumours evolving in the absence or reduced expression of activated ER-α, thus highlighting the need to address in future studies the presence of local growth factors associated to proliferation, including IGF-I and VEGF. The proliferative index observed in neoplastic epithelium is considerably increased compared to normal, healthy endometrial epithelia, supporting the hypothesis that additional molecules other than the ER-β are involved in the regulation of the proliferative pathways in FEA. In the uterus ER-β has been described as a proliferation controller, while in other organs, such the mammary gland and the prostate, ER-β plays a pro-differentiating role [49].

As referred before, a different situation was found concerning PR. Although the tumours also lost PR expression, this was non-significant in both intensity and percentage of labelled cells for the stroma, contrary to the epithelium. Therefore, endometrial cells in FEA retain the ability to respond to progesterone stimulation, but show a reduced ability to respond to estrogens.

The expression of hormone receptors in FEA is still poorly understood, contrasting to the well-studied hormone status in human endometrial carcinomas. It has been recently proposed that the expression of PR changes during tumour progression in endometrial adenocarcinoma [50]. Several mechanisms for progesterone inhibition of endometrial proliferation have been proposed, including inhibition of proliferation through opposing the proliferative effects of estrogen in normal endometrium, which is generally associated with down-regulation of ER-α actions [50] and up-regulation of ER-β, in a manner that is progesterone dose-related [51].

Data on sex steroid receptors obtained in the present study share some resemblance to results from earlier studies in rabbit endometrial adenocarcinomas. Likewise cats, rabbits are an induced ovulation species [52], but, in contrast to cats, rabbits frequently develop endometrial adenocarcinomas, which present two main histological types: papillary adenocarcinoma and tubular/solid adenocarcinomas. In that species, papillary adenocarcinomas are negative for ER-α and PR [52] and Vinci and collaborators (2010) concluded that PR expression was not directly involved in endometrial epithelial carcinogenesis and that such expression was not of prognostic value [53]. On the other hand, in women ER and PR have been established as prognostic markers for endometrial neoplasms [17, 50]. Also, loss of ER-α and PR is associated with markers of aggressiveness such as age, myometrial infiltration and lymph node status [37, 50]. Interestingly, in FEA, the negative status for ER-α expression in the stromal cells was associated with myometrial invasion. Our results suggest that loss of ER-α in FEA may be related to invasive characteristics of the tumour, and further strengthen the need for additional studies on the putative influence of growth factors acting over the proliferation pathways. In women, it was recently proposed that reduced expression of ER-α and PR-A, particularly in neoplastic stromal cells, may be of utmost importance in predicting invasiveness [54]. Additional studies are needed to ascertain this hypothesis in FEA.

Although in endometrial carcinomas of women, ER and PR show significant correlation [37, 38], we did not fund such association in cats. Interestingly, we found that loss of positive cells for PR in the myometrium of FEA was related to a higher nuclear atypia in carcinoma cells. Recently, Tomica et al. (2014) observed lower levels of PR than ER in the myometrium of high-risk human endometrial carcinomas [55]. Also, a relation between cancer cells and the surrounding tissues has been proposed as a necessary event for endometrial normal functioning and carcinogenesis [56]. Our results suggest that myometrial expression of PR may be related to tumour dedifferentiation and that myometrium may crosstalk with epithelial and stromal compartments during tumour progression.

The expression of hormonal receptors is usually used in humans to provide important information for adjuvant hormonal therapy in steroid-responsive tumours. In women, potential effectiveness of hormonal therapy is dependent upon the patient selection based on positive receptor status [57]. Thus, our study sheds light into whether or not the medical treatment would be of choice for most animals with endometrial carcinomas. Nevertheless, it would be of interest to rely on the expression of sex steroids receptors to predict metastasis development. We strongly recommend that the hormonal receptor status of FEA should be determined by the time of histopathological diagnosis.

To the best of our knowledge, this is the first study on Ki-67 (clone MIB-1) expression in feline cyclic and neoplastic endometrium. In normal endometrium, proliferative indexes were higher in the SGE during the FS, while in the LS the higher proliferative indices were found in the DGE. These findings are in agreement with the morphological features that characterize the cycle of glandular development in the species (respectively the branching of the upper area of the glands in the FS and the coiling of the basal glandular area during LS) [40]. In FEA, the proliferative index was remarkably higher than in normal endometrial epithelia, alike the reported in humans [54]. Ki-67 is widely used to assess proliferative activity. In human endometrial carcinomas its expression correlates with the histological grade, depth of myometrium invasion and risk of carcinoma recurrence [58]. Also in women, Ki-67 status is inversely related to hormonal receptor status, particularly in higher grade, ER-α negative, endometrial carcinomas [37, 38, 50]. In this particular feature, FEA may be included in the group of endometrial neoplasias with high proliferation indexes, but with reduced or null expression of ER-α. As discussed before, this is suggestive of the existence of alternative pathways controlling the proliferation in FEA, which needs to be explored in future studies.

Cytokeratins, the largest group of intermediary filament proteins, are an important partner in the renewal and repair of epithelia, by providing rigidity and strength to cell cytoskeleton, while retaining flexibility [59]. Further, CK represent important differentiation markers for different types of epithelia and epithelial tumours [31]. Particularly, the coordinate expression of CK7 and CK20 defines unique subsets of carcinomas [26]. Different types of epithelia show specific patterns of CK expression; CK7 and CK20 are often named as “ductal-type” keratins [60]. Limited knowledge exists on the normal pattern of CK7 and CK20 expression in domestic mammal endometrium. The present study showed that normal endometrial epithelia in cats present a profile CK7+/CK20+ that shows cyclic variation. For CK7, the intensity of immunolabeling remained unchanged for the surface epithelium, despite the decrease observed in the intensity of the glandular epithelia immunolabeling. As for CK20, an increase in the intensity of immunostaining was observed in the LS. Moreover, the overall expression of CK7 was higher compared to that of CK20.

Immunohistochemical expression of CK7 and CK20 has been used in the differentiation of human primary and metastatic tumours of unknown origin [60–62]. Cytokeratin 7 and CK20 are generally confined to epithelia and cell profile for those CK is largely conserved during malignant transformation ([60], reviewed by [63]). Cytokeratin 7 and 20 are potentially potent epithelial differentiation and tumour markers [60] in human and domestic animals. The association CK7+/CK20- is used in humans to prove the endometrial origin of tumours [60, 61].

Results from the present study also show that FEA retain a CK7+/CK20+ phenotype, despite the decrease observed in CK7 expression and a more heterogeneous intensity of labelling on regards to CK20. These findings corroborate previous reports in smaller case series [32]. Notably, conflicting reports exist on the expression of CK7 and CK20 in FEA: Miller et al. 2003, described 3/6 and 4/6 positive FEA respectively for CK7 and CK20 [18]. It has been suggested that CK20 may play a role in facilitating cytoskeleton breakdown and related keratin filament reorganization [64]. Furthermore, the loss of expression of CK20 has been associated to cell dedifferentiation [60]. Myometrial invasion and atypia – histological features commonly associated to invasiveness - were related to a higher percentage of positive cells for CK20 and a lower intensity in CK7 labelling, respectively. Cytokeratins are also involved in multiple signalling pathways beyond their mechanical functions, among epithelial cells or between the epithelial and mesenchymal compartments [60]. These widely complex mechanisms may be related to our findings, but currently we cannot conclude on the putative role of CK in FEA dedifferentiation and invasiveness. Our sample comprised six (25.0 %) animals with clinical history of mammary gland tumour. One should be aware of the possibility of uterine metastasis of mammary gland adenocarcinomas. Moreover, morphological features of both tumours may be indistinguishable. Unlike primary FEA, feline mammary gland carcinomas are generally negative for CK20, maintaining the immunophenotype of the normal mammary gland in this species [32]. Therefore, CK20 may be helpful to distinguish between endometrial primary adenocarcinoma and a metastatic carcinoma in the uterus, as the pattern of expression of CK20 in different carcinomas is preserved in metastasis [65]. Altogether, CK20 might be an important marker for FEA diagnosis in cases of concomitant mammary carcinomas, since CK7 was previously demonstrated in 50 % of feline mammary gland [32]. The results presented herein confirm the positivity of FEA for CK7 reported by Espinosa de los Monteros and co-workers (1999) [32]. Thus, CK7 profile does not seem valuable in differential diagnosis of FEA and a uterine metastasis of a mammary gland tumour.

## Conclusions

Our results show that FEA have a self-hormonal status, different from that observed in normal endometrium. Their loss of expression of ER-α in all endometrial compartments (epithelium and stroma) as well as in myometrium, while retaining PR expression in stroma and myometrium suggests that epithelial proliferation may be determined by alternative pathways possibly involving local growth factors. As expected, proliferative index assessed by Ki-67 immunoreaction is higher in FEA than in normal endometrium.

Importantly, CK20 is regarded herein as a potentially powerful marker for the diagnosis of primary FEA, enabling to differentiate FEA from metastatic disease from mammary gland. Although other molecular studies are indicated to support our findings, determination of the immunohistochemical CK20 profile of uterine tumours in cats may be of utmost importance in the diagnostic routine.

In the present study, we highlight the importance of evaluation both epithelial, stromal and myometrial cells in neoplastic endometrium, comparing such results with normal controls. These compartments are likely to respond in a different way to overall hormonal environment and probably interact with each other. These mechanisms remain unclear and further studies must be performed to clarify these hypotheses.

With this study, we have unveiled some of the molecular events likely involved in feline endometrium carcinogenesis. This will certainly ascertain tumour morphological characterization. Future studies are needed in order to establish clinical outcome of FEA.

## Abbreviations

CK20: Cytokeratin 20
CK7: Cytokeratin 7
DGE: Deep glandular epithelium
ER-α: Estrogen receptor alpha
FEA: Feline endometrial adenocarcinoma
FS: Follicular stage
HPF: High power field (objective 40x)
LS: Luteal stage
OVH: Ovariohysterectomy
PR: Progesterone receptor
SE: Surface epithelium
SGE: Superficial glandular epithelium
VEGF: Vascular endothelial growth factor
